# Evaluating the long-term portrayal of antibiotic resistance in major U.S. newspapers

**DOI:** 10.1186/s12889-023-16203-8

**Published:** 2023-07-12

**Authors:** Allison Way, Maria Bond, Bradley Nanna, Erik S. Wright

**Affiliations:** 1 Department of Biomedical Informatics, University of Pittsburgh School of Medicine, Pittsburgh, PA 15219 USA; 2 Center for Evolutionary Biology and Medicine, Pittsburgh, PA 15219 USA

**Keywords:** Antibiotic resistance, Antimicrobial resistance, Popular press, Media correspondence, Public health, Journalism

## Abstract

**Background:**

Popular media play a critical role in informing the public about antibiotic resistance, which has remained a health concern for over seven decades. Media attention increases the notoriety of antibiotic resistance and shapes the public’s perception of its severity, causes, and solutions. Therefore, it is critical the media accurately portray scientific knowledge that may shape personal and policy responses to antibiotic resistance.

**Methods:**

We analyzed articles from two major U.S. newspapers, The New York Times and Los Angeles Times, from 1940 to 2019 to assess trends in sentiment and lexicon surrounding antibiotic and antimicrobial resistance.

**Results:**

We observed a gradual increase in the number of relevant articles about resistance, although far fewer than other topics with comparable mortality rates. We found a consistently threatening portrayal of antibiotic resistance as a crisis, reflected in the usage of terms such as “superbug” to refer to some pathogens. Governmental agencies responsible for determining antibiotic usage policies were infrequently mentioned in articles. Blame for resistance was almost exclusively attributed to inappropriate antibiotic use, mainly in animals, rather than appropriate uses of antibiotics.

**Conclusions:**

Collectively, our results provide insights into how popular media can more accurately inform the public about antibiotic resistance. Potential changes include increasing news coverage, avoiding fear-mongering, and adequately conveying the multiple uses of antibiotics that can potentiate resistance.

## Background

Antibiotic resistance continues to make headlines in newspapers since becoming a household concern in the 1950s [1]. The World Health Organization categorizes antibiotic resistance as “one of the biggest threats to global health, food security, and development today”. These concerns have motivated initiatives to combat antibiotic resistance, including limitations on antibiotic use and creation of antibiotic stewardship programs within hospitals to better guide clinicians on prescribing practices [2, 3]. The public’s perception of the severity, causes, and solutions to the problem of antibiotic resistance are shaped by mass media [4, 5]. Yet, there is insufficient information about how the media portray antibiotic resistance and how this portrayal may influence the public’s perception. Bridging this gap in knowledge is particularly important given the fact that policies are often shaped by the public’s concerns and the increasing notoriety of antibiotic resistance intensifies pressure for action.

Surveys in multiple countries indicate the general public has a poor understanding of antibiotics [6–10], with a third or more people believing antibiotics can kill viruses. Many people also report confusion about how resistance occurs, stating the body becomes resistant rather than the bacterium [11, 12]. The general lack of understanding about antibiotics could be a major driver of inappropriate antibiotic use, as physicians are more likely to prescribe antibiotics when they believe patients expect them [13–15]. Both patients and clinicians tend to hold a “why not take the risk?“ perception of antibiotic prescribing [16, 17], and this could be the result of insufficient knowledge about the downsides of antibiotic overuse. Common misconceptions about antibiotics might be the result of insufficient or inaccurate information conveyed through news media. In one study of articles from four U.S. newspapers, 42% of articles studied failed to mention how antibiotic resistance occurs [18]. Another study of major U.S. and Canadian newspapers found only 20% of articles mentioned any risk reduction measures [19]. Similarly, a study of Swedish newspapers found only 5% of articles report that antibiotics are specifically for bacterial infections [20, 21].

Media attention towards antibiotic resistance may convey the severity of the problem, assign blame, or suggest solutions. A previous study of media surrounding antibiotic resistance found that crisis type language, such as the phrase “antibiotic apocalypse,“ draws attention to the issue, which may indirectly drive funding, research, and policy in counterproductive ways [22]. Media framing effects can also influence how the public responds to news coverage. A study on the focus of news articles in Australia and the United Kingdom found that alternative framings could encourage debate about solutions to antibiotic resistance or complacency with the problem [23]. Nevertheless, it remains unclear whether media coverage of antibiotic resistance directly impacts the actions of readers and whether this has an effect on resistance rates. News coverage could encourage investment in new antibiotics and the establishment of policies that seek to minimize off-label antibiotic use. Mass media also presents an opportunity to discourage common behaviors that may contribute to resistance, such as self-medicating with antibiotics or pressuring physicians for antibiotics to treat viral infections.

Considering antibiotics are a public good, there are many perspectives about who bears responsibility for antibiotic resistance. Surveys of adults with previous antibiotic use found most people view resistance as being the responsibility of the doctors, researchers, and other patients rather than themselves [11]. Over 70% of antibiotics (by weight) are used in agriculture for pathogen prevention and growth promotion [24–28]. The manure from these farms can contain antibiotics and antibiotic resistant bacteria that may contaminate nearby water sources or carry onto produce farms when used as fertilizer [29–31]. A study on antibiotic resistance and agriculture in three major U.S. newspapers found almost a quarter of the articles analyzed used biased or incorrect information to address the connection between resistance and livestock, leading readers to form inaccurate conclusions [32]. Furthermore, antibiotics used in agriculture may end up in food at low levels, with unknown consequences for consumers [33]. The varied uses of antibiotics and origins of resistance highlight the need for “one health” solutions to the problem [34]. Thus, mass media plays a major role in providing information to help readers keep up-to-date and may even serve a critical role in combatting antibiotic resistance.

Since newspaper articles have a major influence over public opinion and knowledge, we set out to analyze historical sentiment and lexicon surrounding antibiotic resistance. Studying previous news articles about antibiotic resistance is valuable to determine how the discussion has changed over time, when current topics originated, and how media coverage could be improved in the future. We focused on two long-running newspapers in the U.S., The New York Times (NYT) and Los Angeles Times (LAT), which rank in the top four newspapers in the U.S [35]. Both newspapers have substantial numbers of articles available dating back to the time when antibiotics first became widely used in the mid-1940s. The LAT is the largest newspaper not headquartered on the east coast and has a print and online weekly audience of 4.4 million readers [36, 37]. The NYT is predominantly on the east coast and, as of 2021, has almost 8.4 million subscribers [38, 39]. Using news articles originating before the COVID-19 pandemic (the 1940s to 2010s), we analyzed trends in word usage over time and how they reflect historical events. We sought to determine how media could better portray the problem of antibiotic resistance and provide suggestions to this end.

## Methods

Articles were retrieved from ProQuest Los Angeles Times historical archive between 1940 and 2019 and ProQuest The New York Times historical archive between 1940 and 2019. ProQuest encompasses news articles published both in print and online. Articles were required to contain “antibiotic” or “antimicrobial”, as well as “resistant” or “resistance”. Articles and editorials were the only types of items included in this analysis. We manually reviewed and classified the articles into the following categories:

- Broad*: Article covers antibiotic resistance overall in a broad sense, with no specific focus on resistance in the article.
- Narrow*: Article covers antibiotic resistance specifically, with a major focus being specific information pertaining to antibiotic resistance.
- Narrow Pathogen*: Article covers antibiotic resistance specifically, with the focus being the bacterium pertaining to resistance.
- Narrow Drug*: Article covers antibiotic resistance specifically, with the focus being the drug pertaining to resistance.
- Secondary*: Article covers another topic other than antibiotic resistance for the most part, but some information pertaining to antibiotic resistance is included in at least multiple sentences.
- Secondary Meeting: Article covers some form of meeting (e.g., a conference or panel) but includes information with a focus on antibiotic resistance for at least multiple sentences.
- Secondary Development: Article covers the development of a drug but includes information with a focus on antibiotic resistance for at least multiple sentences.
- Marginal: Antibiotic resistance is mentioned in one sentence.
- Irrelevant: Although key words are in the article, they do not occur together, and the article is irrelevant to antibiotic resistance.

*Article types included in analysis

After categorization, optical character recognition was used to convert each news article to plain text. Only articles categorized as broad, narrow, narrow pathogen, narrow drug, or secondary were used for analysis. Word usage was tallied in a term document matrix using the *tm* (v0.7.8) package in R (v4.1.2) [40]. After removing stop words (e.g., “of”), all remaining words underwent stemming and were converted to lower case. Stemming removes prefixes and suffixes of a word to isolate the root, such as removing the “er” at the end of “disaster”. This was done so that all possible forms of a word would be included. A presence/ absence matrix was constructed, representing whether a term appeared in each article. The matrix was then converted into relative word frequency per article by decade, signifying the fraction of articles that used each word one or more times. To apply a data-driven approach to selecting words for analysis, k-means clustering was used to group the articles into 10 groups per news outlet based on their relative word usage. Each news outlet’s clusters were compared to the remaining corpus, and enriched terms from each cluster were manually selected for further investigation. Word stems were insufficiently specific for multiple analyses, which necessitated manual curation of articles. In these cases, we searched for each term in the corpus and manually assessed articles for relevancy to the analysis, as noted below when applicable.

## Results

**Fig. 1:**
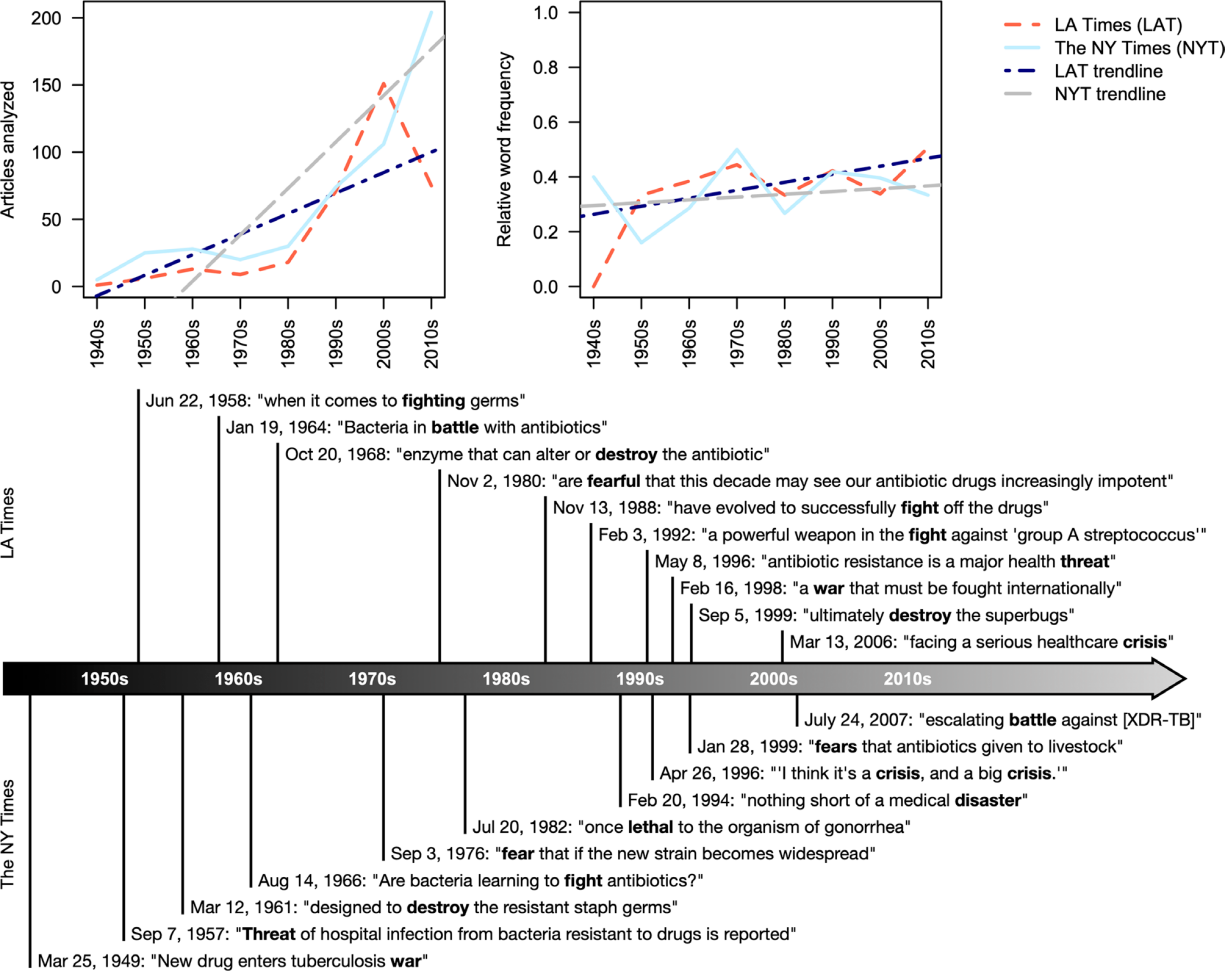
News articles in the NYT and LAT corpuses pertaining to antibiotic resistance increased over time and frequently used threat-related terms. Trendlines show the least squares line of best fit using regression weighted by the number of articles in each decade. Selected quotes containing terms included in the threat dictionary (bold) show that the depiction of resistance as a major threat to the public dates back nearly to the discovery of antibiotics

We began by comparing inter-rater variability across our authors who classified news articles. There were 918 articles in the LAT corpus and 1,629 in the NYT corpus. In total, AW read 904 articles, BN read 1,122 articles, and MB read 650 articles. Of 74 articles independently read by both BN and MB, there was 76% categorical agreement as to whether the article should be analyzed and an interrater reliability of 0.44 across all categories (Krippendorff’s alpha). Among the 47 articles independently read by both AW and MB there was 87% categorical agreement as to whether an article should be analyzed and an interrater reliability of 0.24 across all categories (Krippendorff’s alpha). Given the relatively low interrater reliability, we chose to focus our study on the set of categories deemed relevant rather than analyzing each category separately. The final corpus of analyzed articles consisted of 344 from the LAT and 492 from the NYT (Fig. 1), of which 343 and 451 were convertible to text with optical character recognition, respectively.

### Newspaper coverage of antibiotic resistance is increasing over time and portrayed as a threat

In the span of 80 years across two major US newspapers, 836 articles discussing antibiotic resistance were published (Fig. 1). The Centers for Disease Control and Prevention reported antimicrobial resistant infections affected about 2.8 million people in 2019, with approximately 35,000 dying as a consequence [41]. By comparison, approximately one-third of news coverage is dedicated to crime and there were an estimated 16,425 homicides in 2019 [42, 43]. Likewise, in the span of three months (January to March of 2020), 5,285 articles were published on the COVID-19 pandemic among three major US news outlets [44]. Therefore, although there was a statistically significant increase in publications about antibiotic resistance (two-sided t-test p = 0.11 for LAT and p = 0.003 for NYT), antibiotic resistance still receives far less mass media attention than other causes of death in the U.S.

We used a previously created threat dictionary as a tool for quantifying threat levels in news articles [45]. We manually selected word stems from the threat dictionary that are relevant to antibiotic resistance (“battle”, “crisis”, “destroy”, “disast”, “fear”, “fight”, “lethal”, “suffer”, “threat”, and “war”) to create an overall threat index for each outlet quantifying the relative frequency of articles where any of these words were used. For each word in the threat dictionary, we manually identified the most pertinent occurrence and categorized the use of the word as relevant or irrelevant to resistance within the news article. Averaged across all decades, 35% of analyzed articles in the LAT and NYT contained words from the threat dictionary used in the context of resistance. The threat index increased only marginally over the time period analyzed (Fig. 1), and the increase was not statistically significant (two-sided t-test p = 0.09 for LAT and p = 0.51 for NYT). Excerpts containing words in the threat dictionary show that antibiotic resistance has been described in ominous terms going back to the mid-1900s (Fig. 1). Taken together, these results indicate resistance is portrayed as a threat that is receiving increasing media attention over time.

### Specific pathogens are the focus of news coverage in each decade

**Fig. 2:**
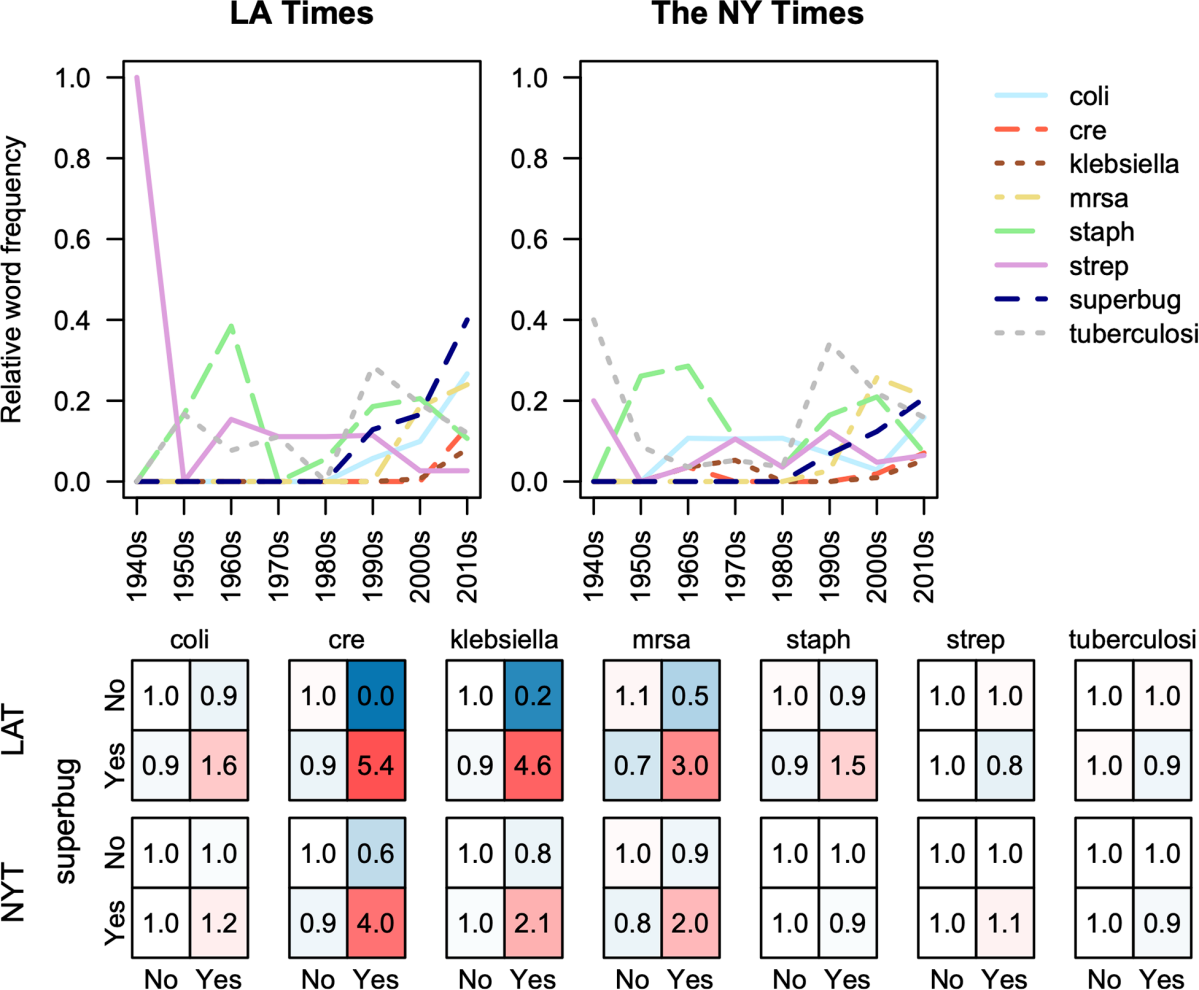
Pathogen word stems display differing levels of news coverage over time. Klebsiella, MRSA, and CRE became associated with the term “superbug” since the term rose to popularity in the 1990s. Fold-difference from the expectation of independence is shown for whether (i.e., “Yes” or “No”) each word stem (pathogen) was used in the same articles as the term “superbug”. Red-shaded boxes represent cases where “superbug” is mentioned with another word stem more frequently than expected and blue-shaded boxes less frequently

Four bacterial pathogens, *Escherichia coli*, *Staphylococcus aureus*, *Klebsiella pneumoniae*, and *Streptococcus pneumoniae*, cause the greatest resistance burden worldwide [46]. We found considerable variability in which pathogens were mentioned in news articles from each decade (Fig. 2). Carbapenem resistant Enterococci (CRE), *Klebsiella*, and methicillin resistant *Staphylococcus aureus* (MRSA) were significantly associated with use of the term “superbug” in individual articles (two-sided Fisher’s exact test p-values 3 × 10^− 8^, 2 × 10^− 4^, and 3 × 10^− 10^ for LAT, respectively; 6 × 10^− 4^, 0.16, and 6 × 10^− 3^ for NYT). None of the other pathogen-related word stems we assessed achieved statistical significance (Fig. 2), implying the term superbug is largely used in reference to CRE, *Klebsiella*, and MRSA.

The first five articles we found about antibiotic resistance were published in the NYT in the 1940s. On June 16th, 1946, an article was published about a scientist attempting to determine how bacteria become resistant to penicillin and sulfa drugs. On November 20th, 1948, there was an article about fear “that the syphilis organism may develop a defense or resistance against penicillin”, although there was no evidence of resistance at that time. On March 25th and 27th, 1949, there were articles published about the discovery of neomycin to treat tuberculosis. The articles noted that neomycin was discovered by Selman Waksman, a co-discoverer of streptomycin, and his student, Hubert Lechevalier. On October 15th, 1949, there was an article about combining streptomycin and penicillin to overcome resistance to penicillin by different pathogens. Collectively, these articles validate the notion that resistance has been a topic in popular media since the 1940s, although the specific antibiotics and pathogens of focus have continued to change.

### Agriculture is a major focus of news coverage on antibiotic resistance

**Fig. 3:**
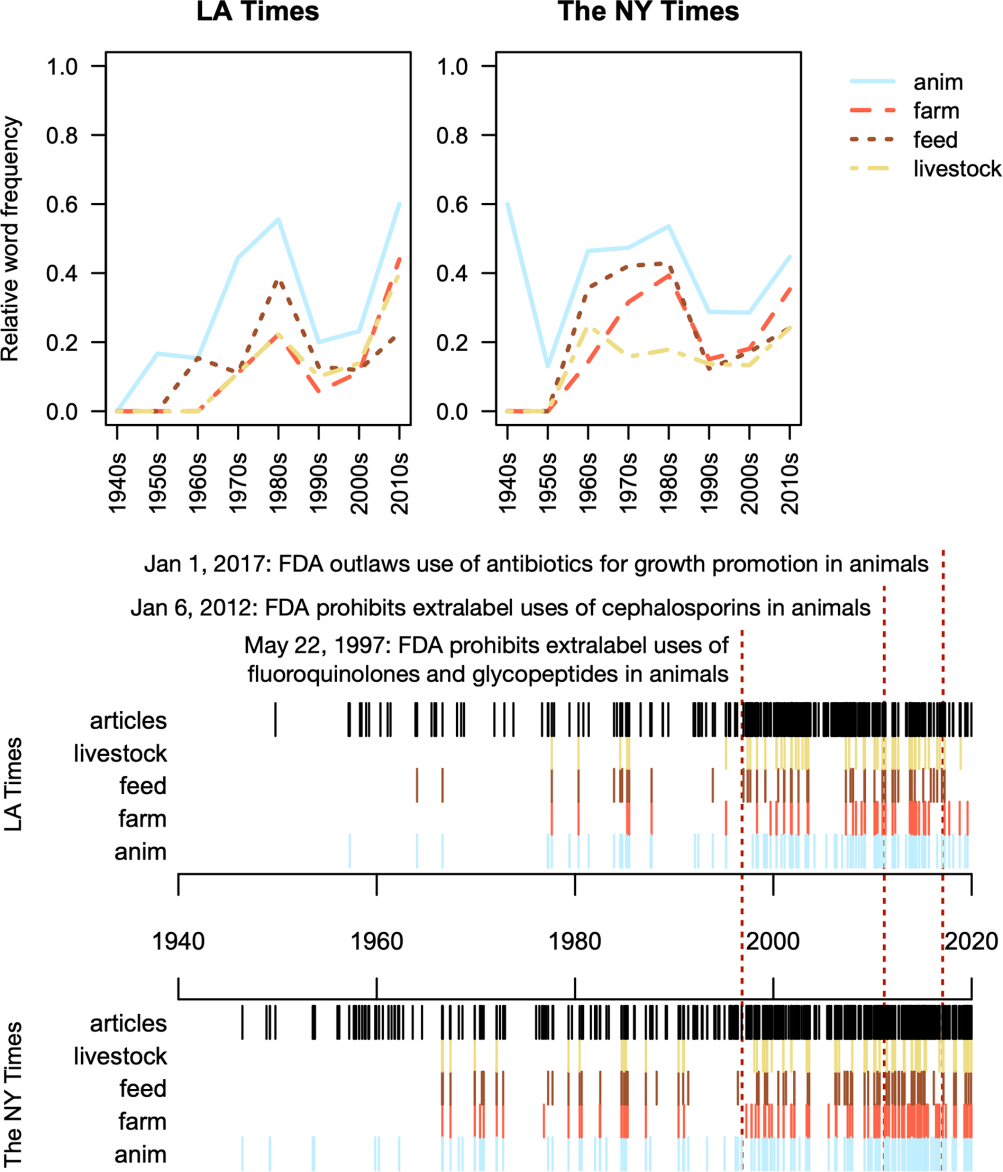
Both news sources showed similar trends in the coverage of agriculture-related word stems, with peaks in the 1980 and 2010s. The frequency of agricultural terms (colored lines) relative to all news articles (black lines) increased with the gradual prohibition of antibiotics for production uses (e.g., growth promotion) in animals

Since the majority of antibiotics are used in agriculture, we analyzed usage of the agriculture-related stemming terms “anim”, “farm”, “feed”, and “livestock” from the 1940s to 2010s (Fig. 3). Most of these terms peaked in the 1980s before decreasing in the 1990s and increased again through the 2010s in both news sources. At its peak, about 50% of articles used the stemming term “anim”, suggesting a focus on antibiotic resistance in agriculture and outbreaks of antibiotic resistance in food. The 1980s saw a push for organic foods where antibiotic use is prohibited, leading to the movement to eliminate antibiotics from farming [47]. In 1997, antibiotics made the headlines again due to the FDA prohibiting the use of extra-label fluoroquinolones and glycopeptides in food-producing animals (Fig. 3). In 2010, the FDA published the first annual summary report of antibiotics used in food-producing animals [48], which was followed by a gradual reduction in antibiotic use in agriculture [49]. It was unclear whether additional news coverage about the use of antibiotics in agriculture was an impetus for policy changes, a result of recurrent policy changes, or both.

### Agencies determining antibiotic policies receive little attention in news articles

Mass media plays a role in informing and reminding the public about antibiotic resistance and what can be done about it. We analyzed usage of agency-related stemming terms “cdc” (Centers for Disease Control and Prevention), “congress”, “doctor”, “fda” (U.S. Food and Drug Administration), “hospit”, “patient”, and “usda” (U.S. Department of Agriculture) to assess which entities are mentioned in articles about antibiotic resistance (Fig. 4). As may be expected, patients, doctors, and hospitals are regularly mentioned in articles from both newspapers. However, we found U.S. governmental agencies were mentioned in only about 10–20% of articles despite their important role in setting policies for antibiotic use [50]. The dearth of articles mentioning the CDC, FDA, or USDA (Fig. 4) relative to articles using agriculture-related word stems (Fig. 3) suggests a minimal focus on policy in newspapers.

**﻿Fig. 4:**
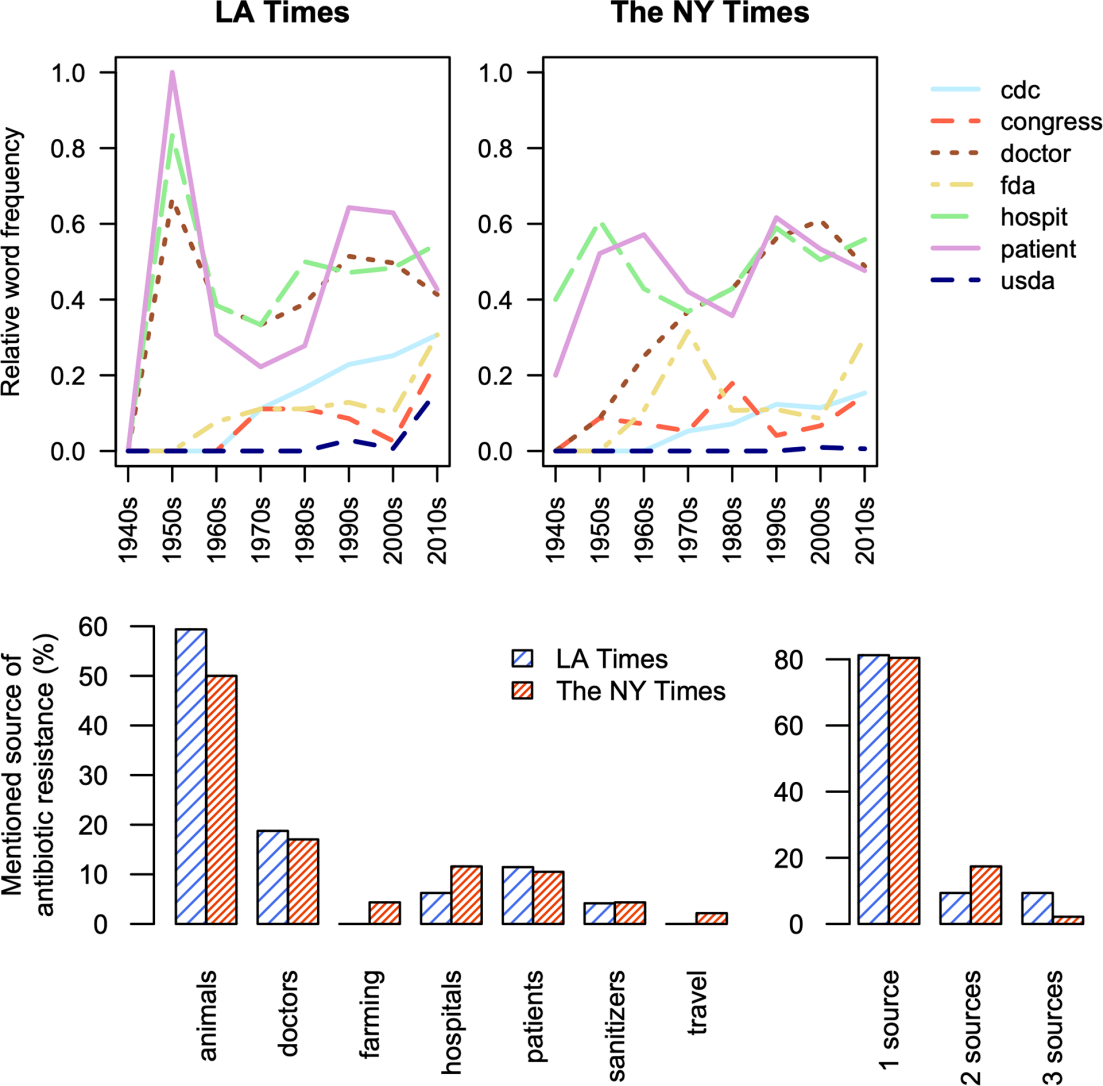
Word stems for medical entities involved in antibiotic resistance (i.e., “doctor”, “hospit”, and “patient”) consistently received more attention than government agencies responsible for policy. Within news articles citing the CDC, FDA, or USDA, antibiotic resistance was most often attributed to overuse of antibiotics in animals for growth promotion and inappropriate prescribing by doctors. The bar chart shows the proportion of instances where resistance was attributed to a source. Instances were split evenly when an article mentioned more than one source of resistance. Note that articles with zero identified sources were excluded

The rarity of mentioning federal agencies could be in part due to the complexity of regulating antibiotics in their dual roles as drugs and growth promoters. In surveying articles, we noticed the CDC was generally referred to as a source of expert information, the FDA was typically mentioned with regard to approving antibiotics for medical and non-medical applications, and the USDA mostly referenced with respect to food safety and outbreaks. There were rarely calls for legislators (i.e., Congress) to act, but these calls often lacked clarity as to what specific actions should be taken. However, there were frequent references to the dearth of new antibiotics and lack of sufficient monetary incentives for antibiotic development, implying a solution would be to incentivize the development of new antibiotics. In contrast to federal agencies, doctors were portrayed as the primary stewards of antibiotics and were noted for their concern or lack of concern towards following prescribing guidelines.

Given the many different stakeholders involved in antibiotic usage, we sought to investigate the assignment of blame for antibiotic resistance. To this end, we manually reviewed the subset of articles (n = 86 and 119 for LAT and NYT, respectively) containing mentions of the CDC, FDA, or USDA for excerpts ascribing resistance to a source. Almost all instances attributed antibiotic resistance to inappropriate use or overuse of antibiotics rather than their appropriate use to treat bacterial infections, although both are likely contributors to resistance evolution. We only found a single article explicitly stating that proper use of antibiotics may result in resistance, although this notion may also be implied by other articles depending upon interpretation. Most commonly, use in animals for production uses (e.g., growth promotion) was blamed for antibiotic resistance (Fig. 4), and a lack of regulation was pointed to as the premier causal factor. Patients were often portrayed as the cause of resistance for not finishing their full course of treatment and doctors for inappropriately prescribing antibiotics, often under noted pressure from patients. Other possible sources of resistance, such as hospital sanitation (e.g., enforcing handwashing), farming (e.g., treatment of citrus blight), or sanitizers (e.g., triclosan in soap), were infrequently blamed for resistance. The finding that blame for resistance is often ascribed to antibiotic use in animals is consistent with the large proportion of news coverage about agriculture (Fig. 3).

## Discussion

Popular press plays an important role in educating, informing, or reminding the public about science issues. To this end, we assembled a set of best practices for reporting on antibiotic resistance. First, the press could report on this issue more frequently. In comparison to other threats and public health concerns, antibiotic resistance has received relatively little attention in the press despite the public playing a role in its control [51]. Second, antibiotic resistance is frequently sensationalized as a crisis of impending doom with threatening rhetoric, possibly because fear-mongering increases readership. However, the available data supports that resistance rates are largely stable in the U.S. overall but might be rising or declining for some pathogens and antibiotics [52]. This punctuated equilibrium stands in sharp contrast to the media’s continual portrayal of antibiotic resistance as a rapidly escalating crisis. In reality, the existence of antibiotic resistance is a problem for many patients even if the rates of resistance are not increasing, because resistance renders drugs ineffective and increases mortality when an ineffective antibiotic is prescribed. One consequence of this continued fear-mongering is that it discredits scientific information over time, causing the public to believe it is not actually a problem. This is also the case when compared to epidemics that are shorter lived and are discussed in terms of fear for a much smaller time frame [53].

The press could more regularly clarify the appropriate uses of antibiotics and origins of antibiotic resistance. Many previous studies revealed a general lack of knowledge among the public about whether antibiotics can cure viral infections and a poor understanding of the fact that antibiotic usage is the premier causal factor in bacterial resistance. A study on the “knowledge deficit model” as it pertains to antibiotic resistance, defines a gap between the public’s knowledge on this topic and the amount they should have to understand antibiotic resistance. The authors suggested the key to bridging this deficit is flooding the public with information, as they are more likely to respond to antibiotic resistance if they are aware of the negative effects [54]. Journalists have an opportunity to regularly inform and remind the public about the science surrounding antibiotics, which may partly mitigate the pressure patients put on their doctors for inappropriate antibiotic prescriptions. In the United Kingdom and Australia, studies found over half of practitioners felt influenced by patients to prescribe antibiotics even when unnecessary [55, 56].

Self-medicating with antibiotics is another potential driver of resistance. There are high rates of antibiotic use without a prescription in the U.S. and other countries [57–59]. Patients may self-medicate with previously unused antibiotics, shared antibiotics, or antibiotics dispensed without a prescription in some cases. Even with a prescription, antibiotic treatment is not without consequences to the individual patient, and these consequences could be clearly conveyed in news articles. Examples of consequences include temporary or permanent changes to the patient’s microbiome, side effects, allergic reactions, and secondary infections such as *Clostridium difficile*. Messaging through antibiotic stewardship efforts is effective at decreasing the improper use of antibiotics, and it is imaginable that messaging through mass media could also have an effect [60, 61]. Nevertheless, it should be noted that the appropriate use of antibiotics also contributes to antibiotic resistance, despite receiving negligible blame in the articles we analyzed. Journalists would be fair-minded to mention that there is no panacea for antibiotic resistance, but there are changes that could lead to improvements over the status quo.

An important limitation of this study is that only US newspapers were studied, limiting the breadth of articles surveyed about antibiotic resistance. We also only included two major newspapers, although our conclusions were supported by both newspapers. The newspapers we included may have exhibited biases that skewed their news coverage, although we suspect antibiotic resistance is largely viewed as a non-partisan issue. Second, the stemming of terms is a limitation, as collapsing disparate words in some cases could artificially inflate the frequency of the word stem. For example, although unlikely in terms of antibiotic resistance, “anim” is being interpreted as “animal” but could also be derived from “animation” after stemming. We only manually curated word usage when word stems were regularly used in contexts that were irrelevant to our analyses (i.e., Fig. 1) or context was required (i.e., the assignment of blame). The word stems we selected are only a subset of the vernacular surrounding antibiotic resistance, and further investigation is possible. Our analyses relied on manual curation of word usage and article categorization, which makes the results more susceptible to unconscious biases, subjective choices, and mistakes. Notwithstanding these limitations, we suspect that the two newspapers studied and our main conclusions are generally representative of the sentiment and lexicon pertaining to discussions of antibiotic resistance in the U.S.

Although it was not the focus of this study, social media continues to grow as a medium for journalism and has the potential to reach wider populations more quickly than traditional news media [62]. Social media also offers the prospect of better disseminating educational information about antibiotics to low- and middle-income countries [63]. Many researchers, government agencies, and physicians have employed social media to inform their social networks. Nevertheless, popular newspapers can still reach enormous audiences through a combination of traditional articles and their millions of followers on social media. While social media holds the promise of incorporating more experts and diverse perspectives, the interactive nature of social media may amplify the spread of misinformation that indirectly leads to overprescribing antibiotics [64]. Future research on media coverage of antibiotic resistance may consider the role of social media on the public’s understanding of antibiotics.

Antibiotics researchers, such as ourselves, could do a better job of interfacing with journalists to provide up-to-date scientific information. The main sources of resistance and the methods that can be used to reduce resistance are continually being studied, which presents an opportunity to better inform the public of potential solutions. In particular, there is an opportunity for journalists to work with scientists to indirectly influence public policy through new outlets. Since antibiotics are a public good, any solutions to the problem of resistance are likely to involve multiple stakeholders including pharmaceutical businesses, patients, doctors, insurers, farmers, veterinarians, researchers, and policy makers. Bills are regularly introduced in the U.S. that present measures for combating antibiotic resistance (e.g., the PASTEUR Act), and journalists play a key role in disseminating information that leads to support for these bills. It is our hope that increased interactions between journalists and scientists will contribute to sustainable solutions to the problem of antibiotic resistance and, thereby, better curtail infectious diseases. The COVID-19 pandemic increased connections between journalists and infectious disease specialists that we anticipate are transferable to future news articles written about antibiotic resistance.

## Conclusion

The competing interests of different stakeholders involved in antibiotics makes the problem of resistance challenging to fairly or succinctly portray in popular media. Since antibiotics are a public good, stakeholder perspectives are often at odds and journalists have the difficult challenge of balancing these perspectives when talking about antibiotic resistance to a wide readership. Patients often understandably want antibiotics as a potential cure for their ailment, although this increases the possibility they will no longer work for the next patient. Doctors are torn between their current patients and the need for judicious use to maintain effectiveness for future patients. Meanwhile, agriculture employs antibiotics for various reasons, and these antibiotics may indirectly cause clinically-relevant resistance in human pathogens even if they are different antibiotics due to cross-resistance. Pharmaceutical companies must decide how much to prioritize developing new antibiotics given their predicted market size and profitability, which are partly related to the cost and effectiveness of currently available antibiotics. Regulators are tasked with imposing constraints to balance these competing perspectives, and legislators are expected to promote solutions to antibiotic resistance while being lobbied from different perspectives.

This study revealed historical trends in how journalists have represented the competing forces surrounding the problem of antibiotic resistance. The standard news narrative was that an absence of new antibiotic development, lack of regulation, and overuse in different contexts were driving antibiotic resistance. The main findings of this study are that threatening rhetoric about resistance dates back almost to the discovery of antibiotics, “superbug” recently became part of the common vernacular for describing specific bacteria, agriculture is a major focus of resistance coverage in newspapers, and overuse of antibiotics in animals is commonly blamed for resistance. Our study provides an overarching perspective on the way resistance was historically portrayed to the public through U.S. newspapers up until the arrival of SARS-CoV-2. How COVID-19 shaped discourse around antibiotic resistance could be a topic of future inquiry. Journalists have the critical role of helping to disseminate news and knowledge about resistance to a wide audience, and we hope the conclusions of this study will further refine the perspective of journalists writing news articles about antibiotic resistance. As several news articles stated, antibiotics are not a “silver bullet” for bacterial infections, and we note there is no “silver bullet” for antibiotic resistance. Journalists play an important, albeit indirect, role in countering resistance, and the complexity of the problem demands a group effort to work towards solutions.

## Data Availability

The data that support the findings of this study are under copyright and are not publicly available. Analysis code will be shared upon reasonable request to the corresponding author.
